# Right adrenal metastasis from left apical non-small cell lung carcinoma with contiguous tumor thrombus extending through the inferior vena cava into the right atrium

**DOI:** 10.1016/j.radcr.2026.03.060

**Published:** 2026-05-09

**Authors:** Alexander M. Satei, Odai El-Samawi, Bilal Turfe, Tess Vindischman, Daniel Parks, Eric C. Ferguson

**Affiliations:** a Trinity Health Oakland Hospital, Pontiac, MI, United States; b Wayne State University School of Medicine, Detroit, MI, United States; c Huron Valley Radiology, Ypsilanti, MI, United States

**Keywords:** Non-small cell lung cancer, Adrenal, Metastasis, Inferior vena cava, Cardiac chamber

## Abstract

A 76-year-old male presented to our emergency department with intermittent exertional chest pain. Computed tomography of the abdomen and pelvis demonstrated a heterogeneous right adrenal mass measuring 7.7 cm with direct tumor extension into the inferior vena cava (IVC) and right atrium; there was expansion of the IVC lumen suggestive of tumor thrombus. Magnetic resonance imaging confirmed invasion of the IVC with contiguous tumor thrombus extending into the right atrium and spanning approximately 15.5 cm craniocaudally. Image-guided biopsy of the adrenal mass confirmed metastatic poorly differentiated non-small cell lung carcinoma (NSCLC). Subsequent computed tomography of the chest demonstrated a large left apical lung mass representing the primary malignancy. After multidisciplinary evaluation, the patient elected hospice care. Adrenal metastasis from non-small cell lung carcinoma with contiguous intravascular tumor extension into the IVC and right atrium is exceedingly rare. Our case highlights this uncommon pattern of NSCLC metastasis, including incidence, clinical presentation, imaging characteristics, management, and prognosis.

## Introduction

Lung cancer remains the leading cause of cancer-related mortality worldwide, with non-small cell lung carcinoma (NSCLC), including adenocarcinoma, squamous cell carcinoma, and large cell carcinoma subtypes, accounting for approximately 85% of cases [1]. Metastatic spread is common at the time of diagnosis, most commonly involving bone, the central nervous system, liver, and adrenal glands [2]. Adrenal metastases occur in up to 20%-45% of patients with lung cancer in autopsy series and typically present as isolated nodules confined to the gland [3]. However, extension of metastatic NSCLC from the adrenal gland into the inferior vena cava (IVC) and cardiac chambers is exceedingly rare, only being reported in isolated case reports, and represents an aggressive pattern of spread.

## Case report

A 76-year-old male presented to our emergency department from his cardiologist’s office for evaluation of intermittent exertional chest pain. He described 1 week of chest discomfort radiating to the back, associated with shortness of breath and 2 syncopal episodes. His past medical history was notable for chronic obstructive pulmonary disease, hypertension, chronic pain disorder, and prior episodes of syncope. Initial laboratory evaluation demonstrated leukocytosis (white blood cell count: 19.0 × 10³/µL), anemia (hemoglobin: 10.3 g/dL), and an elevated B-type natriuretic peptide (BNP) of 384 pg/mL. Electrocardiography demonstrated a normal sinus rhythm without acute ischemic changes.

**Fig. 1:**
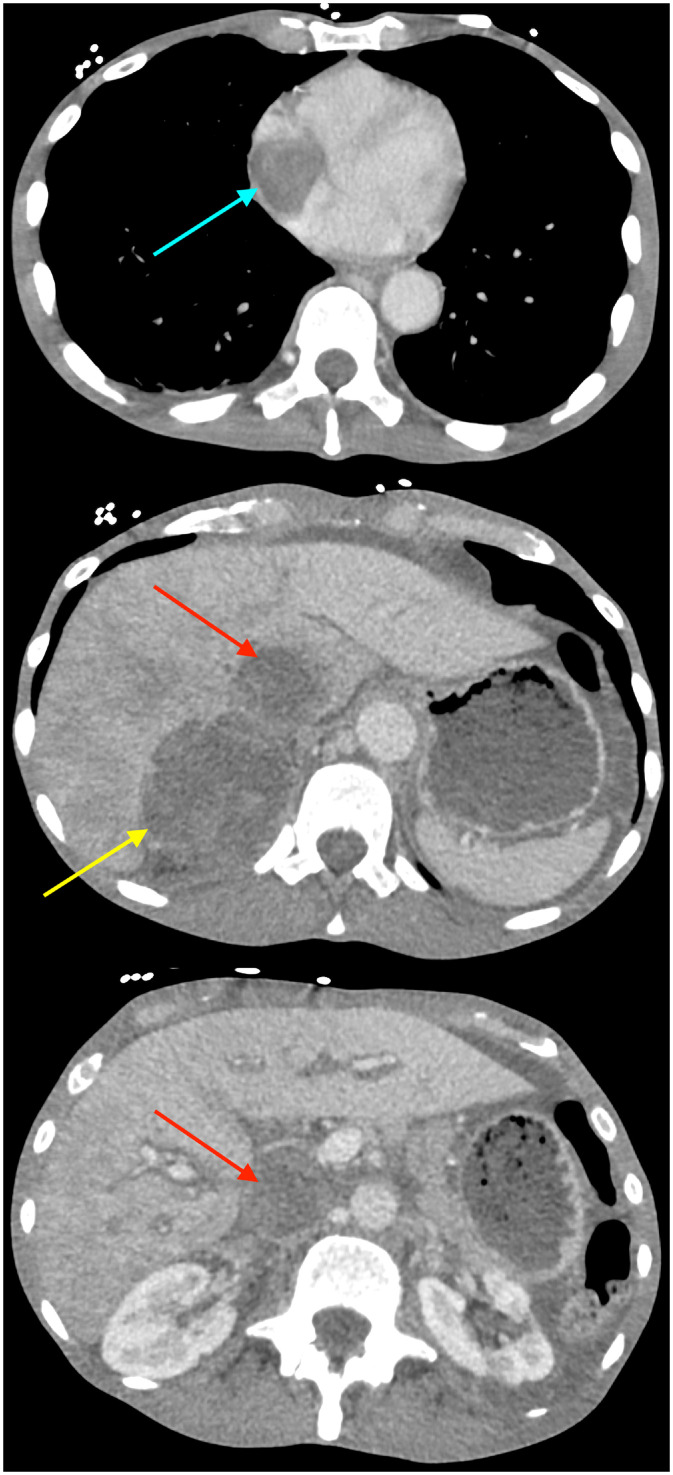
Computed tomography of the abdomen and pelvis with contrast (venous phase) in axial view demonstrates a 7.7 cm right adrenal mass (yellow arrow) with intravascular tumor invasion into the inferior vena cava (red arrow) and right atrium (blue arrow).

**Fig. 2:**
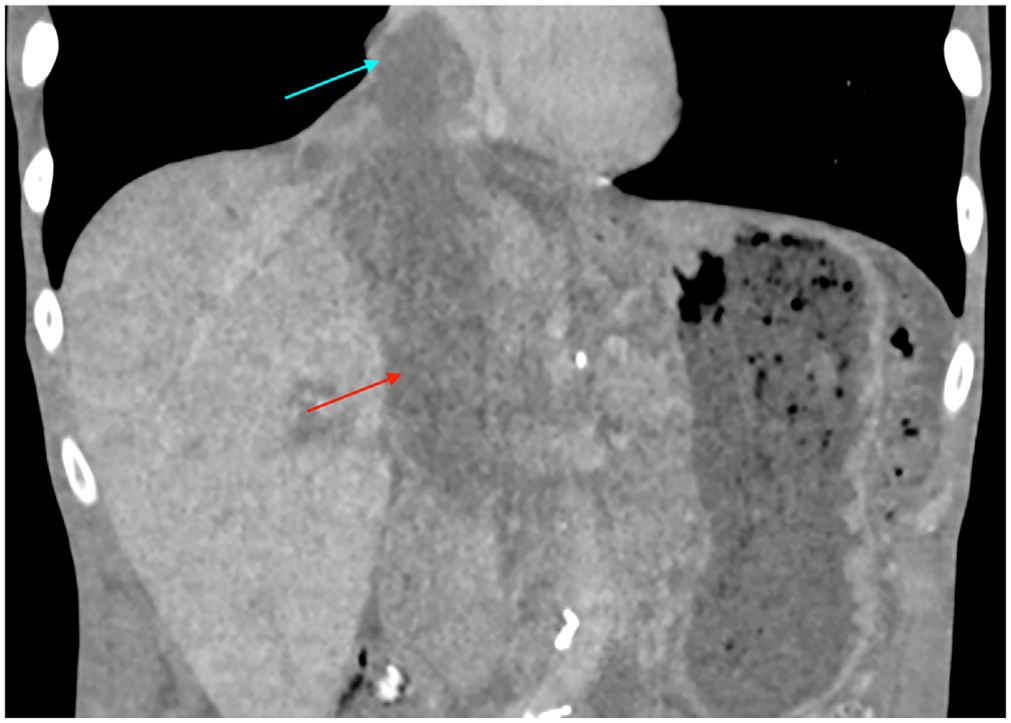
Computed tomography of the abdomen and pelvis with contrast (venous phase) in coronal reformat demonstrates the extent of the inferior vena caval (red arrow) and right atrial (blue arrow) involvement.

**Fig. 3:**
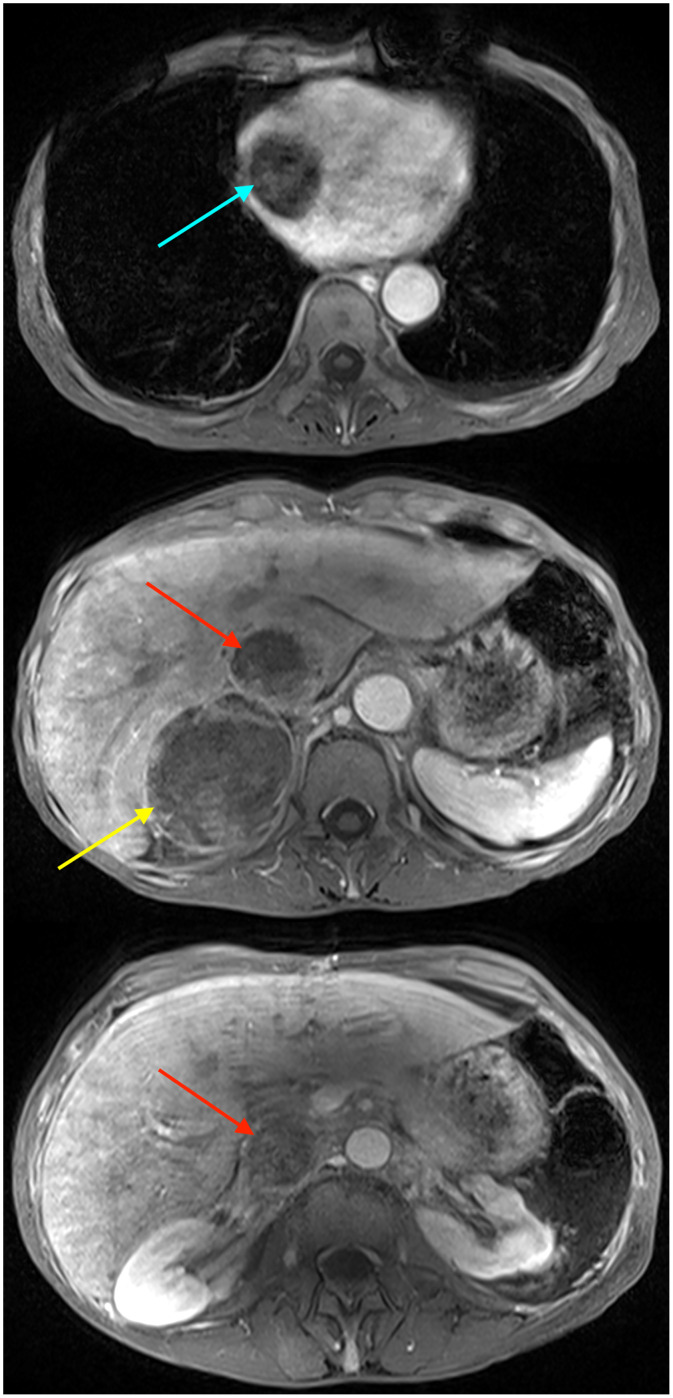
Magnetic resonance imaging of the abdomen with contrast (70 second delay) in axial view demonstrates a 8.2 cm right adrenal mass (yellow arrow) with intravascular tumor invasion into the inferior vena cava (red arrow) and right atrium (blue arrow).

**Fig. 4:**
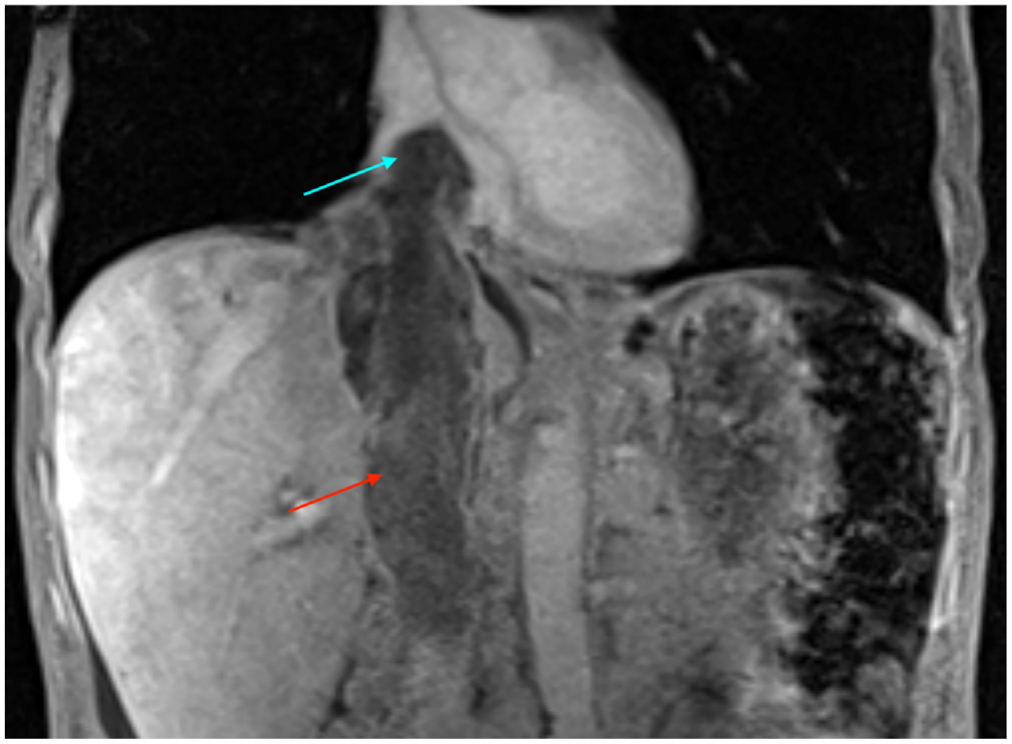
Magnetic resonance imaging of the abdomen with contrast (70 second delay) in coronal view demonstrates the extent of the inferior vena caval (red arrow) and right atrial (blue arrow) involvement.

**Fig. 5:**
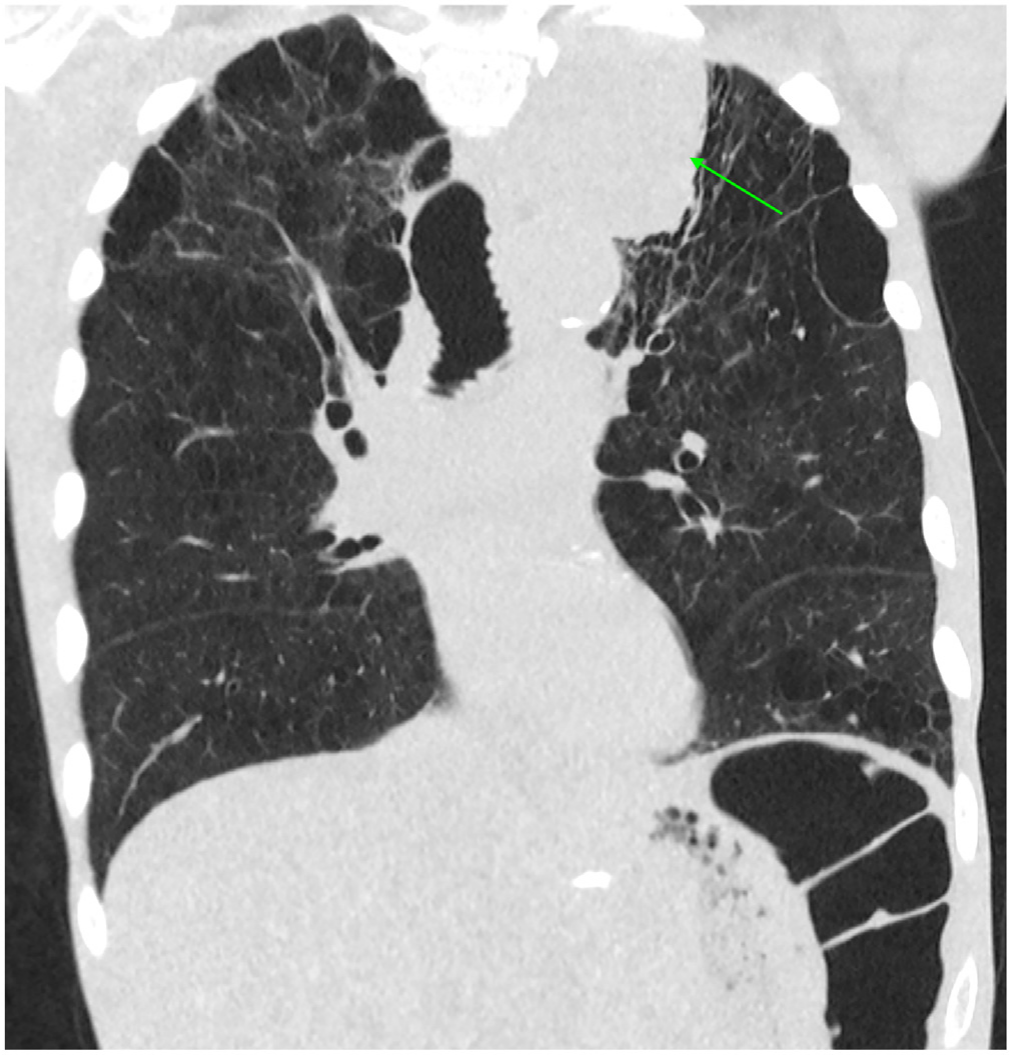
Computed tomography of the chest without contrast in coronal reformat demonstrates a 6.8 cm left apical lung mass (green arrow) representing the patient’s primary site of malignancy.

Contrast-enhanced computed tomography (CT) of the abdomen and pelvis in the portal venous phase revealed a large heterogeneous right adrenal mass measuring 7.7 cm with direct intravascular tumor extension into the inferior vena cava; the tumor expanded the IVC lumen, extended inferiorly to just below the renal veins, and coursed superiorly into the right atrium (Fig. 1, Fig. 2). Magnetic resonance imaging (MRI) of the abdomen with contrast, including T1-weighted, T2-weighted, diffusion weighted, and dynamic post-contrast sequences, confirmed an 8.2 cm right adrenal mass, with invasion of the suprahepatic, intrahepatic, and infrahepatic IVC and right atrium; tumor thrombus spanned at least 15.5 cm craniocaudally and demonstrated post-contrast enhancement on MRI, supporting tumor thrombus rather than bland thrombus (Fig. 3, Fig. 4). Differences in craniocaudal length measurements are likely attributable to variations between imaging modalities. Transthoracic echocardiography demonstrated a large, spherical, fixed mass within the right atrial cavity measuring 4.8 × 3.8 cm, corroborating the intracardiac extension seen on cross-sectional imaging. Echocardiography also revealed mildly reduced left ventricular systolic function with an ejection fraction of 40%-45% and abnormal left ventricular diastolic function. CT-guided biopsy of the right adrenal mass demonstrated metastatic poorly differentiated NSCLC, not otherwise specified. Immunohistochemistry was positive for pankeratin and negative for TTF-1, Napsin A, p40, chromogranin, and synaptophysin. Subsequent CT of the chest without contrast identified a large left apical pulmonary mass measuring 6.8 cm invading the left superior mediastinum, favored to represent the site of primary malignancy (Fig. 5).

The initial imaging differential for an adrenal mass with extensive venous invasion included adrenocortical carcinoma, renal cell carcinoma with venous extension, and metastatic disease. Renal cell carcinoma was considered unlikely given the mass was centered in the adrenal gland and no discrete renal mass was identified. Metastatic disease and primary adrenocortical carcinoma remained the leading considerations; however, metastatic disease would become the favored diagnosis after CT of the chest revealed a large left apical pulmonary mass.

## Discussion

Tumor involvement of the IVC is most classically seen with renal cell carcinoma, although can be less frequently observed in cases of hepatocellular carcinoma, adrenocortical carcinoma, and primary leiomyosarcoma [4]. Direct vascular or cardiac chamber invasion is rarely observed in cases of NSCLC, with literature limited to a handful of case reports and case series [5]. Furthermore, contralateral adrenal gland metastasis without ipsilateral involvement is relatively uncommon, with one case series suggesting an incidence of 30% in a cohort of 50 patients with NSCLC with confirmed metastasis to the adrenal glands [6]. In our case, metastatic NSCLC from a primary left apical mass both involved the contralateral adrenal gland and propagated into the IVC and right atrium.

Malignant invasion of the IVC may manifest with abdominal or back pain, lower extremity edema, ascites, and the development of venous collaterals due to impaired venous return [7,8]. When tumor burden extends to the right atrium, patients may experience dyspnea, fatigue, chest discomfort, or syncope, along with signs of right-sided heart failure [9]. Atrial involvement also increases the risk of arrhythmias and embolic events [9].

Cross-sectional imaging plays an important role in evaluating adrenal metastases and defining the extent of disease. CT is typically the initial modality employed and can help differentiate benign from malignant adrenal lesions. Metastasis from NSCLC often appears as a heterogeneous and unilateral adrenal mass [10]. MRI offers superior soft-tissue characterization and is particularly useful for distinguishing malignant tumor thrombus from bland thrombus based on post-contrast enhancement within the thrombus, expansion of the involved vessel, and direct continuity with the primary mass, imaging features demonstrated in our case. Bland thrombus lacks internal enhancement and does not expand the vessel lumen, although partial volume effects, slow flow, and mixed tumor/bland thrombus can limit differentiation [11].

In our case, the size of the adrenal mass and the presence of extensive vascular invasion allowed for confident identification of malignancy on the initial CT, even before the primary lung tumor was known. Fluorodeoxyglucose (FDG) F-18 PET/CT scan aids in assessing metastatic burden, with adrenal metastases and primary adrenocortical carcinoma commonly demonstrating markedly increased uptake, often with maximum standardized uptake (SUVmax) values exceeding 10 [12].

Management of metastatic NSCLC with adrenal involvement typically involves systemic therapy, including chemotherapy or immunotherapy [13]. Radiation therapy may be used for palliative control or for symptomatic venous obstruction. Surgical resection of adrenal metastases is considered in selected patients with isolated metastatic disease. However, intravascular extension into the IVC or cardiac chambers often precludes operative management due to high morbidity and limited survival benefit [13]. In cases with advanced vascular tumor thrombus, such as in our case, therapy is often limited to symptomatic management rather than aggressive oncologic and surgical intervention.

Prognosis in NSCLC with adrenal metastasis varies widely depending on the extent of metastatic disease involvement, with improved survival seen in patients with isolated adrenal involvement compared to those with disseminated disease [14]. Raz et al. found that 5-year overall survival in patients with isolated adrenal metastasis was 34% in those treated with adrenalectomy compared to 0% in patients treated non-operatively [14]. Vascular and cardiac chamber involvement in cases of metastatic NSCLC correlates with significantly worse prognosis [15]. In our case, the patient was ultimately discharged to hospice care after consultation with the oncology and palliative care teams.

## Conclusion

NSCLC commonly metastasizes to the adrenal glands, but contralateral adrenal metastasis with direct extension into the IVC and right atrium is exceedingly rare. Recognizing intravascular tumor thrombus can significantly alter staging, prognosis, and management. Cross-sectional imaging, including CT, MRI, and PET/CT plays a crucial role in identifying malignant vascular invasion and additional sites of metastatic disease. Treatment of advanced NSCLC with vascular and cardiac chamber involvement typically relies on systemic therapy, although prognosis remains poor. Radiologists should be familiar with this rare manifestation to ensure timely and accurate diagnosis and to support multidisciplinary decision-making aimed at maintaining patient quality of life.

## Patient consent

Informed consent was obtained from the patient for publication of this report and accompanying images.
